# Thermo-mechanical contact problems and elastic behaviour of single and double sides functionally graded brake disks with temperature-dependent material properties

**DOI:** 10.1038/s41598-019-51450-z

**Published:** 2019-10-25

**Authors:** Mehdi Bayat, Ibrahim M. Alarifi, Ali Akbar Khalili, Tarek M. A. A. El-Bagory, Hoang Minh Nguyen, Amin Asadi

**Affiliations:** 1 Department of Civil Engineering, Aalborg University, Aalborg, Denmark; 2 Department of Mechanical Engineering, University of Malaya, 50603 Kuala Lumpur, Malaysia; 3 Department of Mechanical and Industrial Engineering, College of Engineering, Majmaah University, Al-Majmaah, 11952 Riyadh, Saudi Arabia; 4 Division of Computational Physics, Institute for Computational Science, Ton Duc Thang University, Ho Chi Minh City, Vietnam; 5 Faculty of Electrical and Electronics Engineering, Ton Duc Thang University, Ho Chi Minh City, Vietnam; 6 Department of Mechanical Design, Faculty of Engineering Materia, Helwan University, Cairo, Egypt

**Keywords:** Mechanical engineering, Computational methods

## Abstract

A thermo-elastic contact problem of functionally graded materials (FGMs) rotating brake disk with different pure brake pad areas under temperature dependent material properties is solved by Finite Element Method (FEM). The properties of brake disk change gradually from metal to ceramic by power-law distribution along the radial direction from the inner to the outer surface. Areas of the pure pad are changing while the vertical force is constant. The ratio of brake pad thickness to FGMs brake disk thickness is assumed 0.66. Two sources of thermal loads are considered: (1) Heat generation between the pad and brake disk due to contact friction, and (2) External thermal load due to a constant temperature at inner and outer surfaces. Mechanical responses of FGMs disk are compared with several pad contact areas. The results for temperature-dependent and temperature-independent material properties are investigated and presented. The results show that the absolute value of the shear stress in temperature-dependent material can be greater than that for temperature-independent material. The radial stress for some specific grading index (*n* = 1.5) is compressive near the inner surface for double contact while it is tensile for a single contact. It is concluded that the radial strain for some specific value of grading index (*n* = 1) is lower than other FGMs and pure double side contact brake disks.

## Introduction

functionally graded materials (FGM) are considered as a new class of inhomogeneous composite materials which are made of two or more different materials usually metal and ceramic^1–5^. FGMs are used in the high-temperature environment where it is not possible to use traditional composite material^6^. The material which gives the high-temperature resistance to FGMs is ceramic because of its low thermal conductivity^7^. The properties of FGMs change from the metal surface to the ceramic surface smoothly and continuously. The microscopic ceramic component of FGMs has excellent heat resistance, and the metal part can withstand toughness and strength. The concept of FGMs was originated in Japan in the mid-1980s during a research project by space scientists to develop thermal barrier material. There are several applicable opportunities for using FGMs across different industries^6,8,9^. The computer circuit, aerospace and airplane industries will benefit from this type of material.

Bayat *et al*.^10,11^ presented the mechanical and thermo-mechanical response of variable FGMs rotating disks by applying the semi-analytical method and exact solution, respectively. Moreover, Bayat *et al*.^12–14^ applied first order shear deformation theory to present the mechanical and thermo-mechanical bending in FGMs rotating disk as well as large deformation in terms of von Karman theory. Afsar and Go^15^ focused on a FGMs thin circular rotating disk under thermal load and solved it by finite element method (FEM). They found that the effective parameters for a FGMs disk are radial disk thickness, temperature distribution profile, the temperature difference of the outer and inner surface, and angular velocity. Nie and Batra^16^ analyzed axisymmetric variable thickness rotating disk with the material properties such as thermal expansion coefficient, shear modulus, and mass density varying along the radial axis. They concluded that the non-homogeneous material properties play an important role in thermo-mechanical results. Hosseini Kordkheili and Naghdabadi^17^ applied mathematical modeling to find the thermo-elastic solution for FGMs solid rotating disks with the axisymmetric situation. Results obtained with linear algebraic equations were compared with one solved by FEM. Peng and Li^18^ analyzed the thermo-elastic problem of a FGMs hollow circular rotating disk with constant angular velocity and steady state thermal load. They showed the effects of change in temperature, gradient parameter, thickness, and angular velocity on the thermo-mechanical responses.

Contact problem is one of the critical issues in rotating subject such as brake disk. Siroux *et al*.^19^ studied the thermal behavior of the grey cast iron disk during periodic sliding contact with an organic matrix composite pin in a braking situation which is employed in railway. Panier *et al*.^20^ conducted an experimental study to explain and classify the appearance of the thermal gradients of hot spot occurrence on the surface of the brake disk. They found pad contact length and pad stiffness are parameters which influence the hot spots development. Anders Söderberg and Sören Andersson^21^ presented the three dimensional model of brake pad and rotor to obtain the pressure in contact areas between pad and rotor by applying FEM. Kermc *et al*.^22,23^ conducted an experimental study on a brake disk system with two different materials as brake disk against same brake pad, the first one was conventional grey cast iron and the second one was a carbon-ceramic composite which was applied against a metal-matrix composite. They found that the contact pressure, rubbing speed, and temperature are influenced by the wear and the friction coefficient of the brake disk and the contacting pad. Shahani and Sharifi^24^ applied nonlinear contact element to investigate the concentration factors and contact stress of a drill pipe by using 3D FEM commercial software such as ABAQUS 6.6.

Contact problem is investigated in FGMs structure. The friction between each layer of FGMs structure is studied. Ke and Wang^25^ investigated sliding friction contact in sub-layers FGMs structure under perpendicular and parallel forces while the Coulomb type of friction was considered. Jang and Ahn^26^ solved a transient FEM contact problem for stationary FGMs between sliding layers under two-dimensional thermo-elastic instability condition as a result of frictional heating at the interface. They concluded that FGMs control the development of chaos and delay the separation. Elloumi *et al*.^27^ obtained contact stresses and stick zone friction coefficient by solving two-dimensional partial slip contact problem between a rigid punch and a non-homogeneous half-plane while the material property changed exponentially. Barik *et al*.^28^ applied Fourier integral transform to investigate stationary FGMs plane contact problem between the punch and a half-space of the rigid insulated body when the gliding of punch and half-space was the source of heat generation. Lee and Jang^29^ studied the effect of FGMs half plane sliding with constant velocity against a homogeneous conducting body under thermo-elastic instability. Jip Choi and Paulino^30^ analyzed the contact problem and thermo-elastic situation graded coating fixed surface on sliding rigid flat. Shahzamanian *et al*.^31^ solved the thermo-elastic contact problem with FEM in a FGMs rotating brake disk. They considered heat source due to friction which generated during contact between brake disk and different pad thickness. They found the ratio of pad thickness to FGMs brake disk thickness for full-contact status. Lee and Jang^32^ investigated thermo-elastic instability due to the friction effect in the system with a layer of FGMs sliding between two half-plate homogeneous materials with constant velocity. It is needed to highlight that the present analysis is simplified as the effect of losing mass and wearing are not taken into account; more details can be found in^33,34^. Meanwhile, the effect of thermo-elastic instability, which is the results from the interaction of frictional heat generation, thermoelastic distortion, and elastic contact, is known as frictionally excited thermoelastic instability^33,34^. It generally leads to the establishment of localized high-temperature contact regions known as hot spots. This topic is out of the aim of this study. In thermo-elastic instability, several parameters such as thickness, velocity and other parameters will be investigated. In this regard, further detail can be referred to^35–37^ and references therein. Here, the effect of varying the material properties with position and temperature are considered. Here, the effect of FGMs is covered.

**Figure 1:**
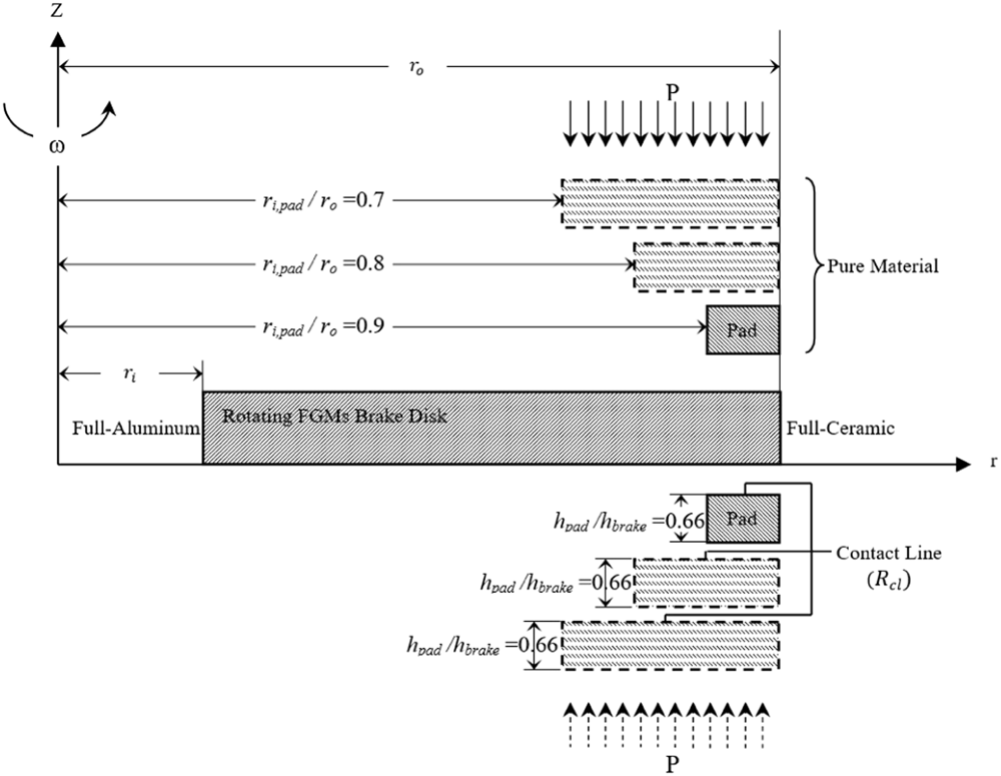
Configuration of brake and double contact sides.

Based on the authors’ knowledge, no work has been reported to demonstrate the effect of geometry, temperature-dependent material property, and gradation of material properties for FGMs. The thermo-mechanical analysis of double side contact problem for FGMs brake disk and pure pads is also not discussed. In this paper, FEM is employed for solving temperature-dependent material properties of the rotating FGMs brake disk with double side pure pads for different contact areas, as shown in Fig. 1. The brake disk is composed of a ceramic and aluminum at outer and inner surfaces, respectively. The material properties change in a radial direction by power law distribution. Three different contact areas between the pad and FGMs brake disk are considered. Constant vertical forces on pads are assumed. For all different cases contact area, the thickness ratio of the pad to brake disk is assumed 0.66. Friction between pads and brake disk produces thermal load. The temperatures are assumed as 100 °C at the inner surface and 25 °C at outer.

## Temperature Dependent Material Property and Gradation Relation the Different Contact Areas

The properties (*P*) of FGMs brake disk change along the radial direction by the power-law distribution^7^:

1 $$P(r)=({P}_{o}-{P}_{i}){(\frac{r-{r}_{i}}{{r}_{o}-{r}_{i}})}^{n}+{P}_{i}$$

Where *P* and *r* are material property and radius of FGMs brake disk, respectively. The subscript *i* and *o* represent the value of relathe ted character at inner and outer surfaces, respectively. *n* is the grading index in this formula and should be more than 0 ($n$ ≥ 0). Because FGMs are used in high-temperature situation, the temperature dependent material property (*P*)^7^ is:

2 $$P(r,\,T)=({P}_{o}(T)-{P}_{i}(T)){(\frac{r-{r}_{i}}{{r}_{o}-{r}_{i}})}^{n}+{P}_{i}(T)$$

where *P*_*i*_(*T*) and *P*_*o*_(*T*) are calculated from Shen^6^:

3 $${P}_{j}={P}_{0}({P}_{-1}{T}^{-1}+1+{P}_{1}T+{P}_{2}{T}^{2}+{P}_{3}{T}^{3})$$

where *j* = *i* or *o* and *T* shows the absolute temperature (in K). *P*_0_, *P*_−1_, *P*_1_, *P*_2_, and *P*_3_ are the coefficients of *T* which are constant for the constituent material.

In this research, the coefficient of thermal expansion and the modulus of elasticity are calculated by employing the gradation relation (2) from^7^ as

4 $$\alpha (r,\,T)=({\alpha }_{o}(T)-{\alpha }_{i}(T)){(\frac{r-{r}_{i}}{{r}_{o}-{r}_{i}})}^{n}+{\alpha }_{i}(T)$$

and

5 $$E(r,\,T)=({E}_{o}(T)-{E}_{i}(T)){(\frac{r-{r}_{i}}{{r}_{o}-{r}_{i}})}^{n}+{E}_{i}(T)$$

The Poisson’s ratio $\upsilon$ is considered constant along the radial direction for rotating FGMs disk^31^ but for the other material properties of FGMs disk including the density *ρ*, the coefficient of friction *μ*, and the thermal conductivity *k* are considered to be temperature-independent and are calculated by applying Eq. (1) as

6 $$\rho (r)=({\rho }_{o}-{\rho }_{i}){(\frac{r-{r}_{i}}{{r}_{o}-{r}_{i}})}^{n}+{\rho }_{i}$$

7 $$\mu (r)=({\mu }_{o}-{\mu }_{i}){(\frac{r-{r}_{i}}{{r}_{o}-{r}_{i}})}^{n}+{\mu }_{i}$$

and

8 $$k(r)=({k}_{o}-{k}_{i}){(\frac{r-{r}_{i}}{{r}_{o}-{r}_{i}})}^{n}+{k}_{i}$$

## Simulation of Double FGMs Brake Disk by FEM

Double FGMs brake and pure pad disks are simulated by finite element software ANSYS 11 and two brake pads contact on the top and bottom surfaces of the brake disk. Heat is produced due to friction from vertical pressures applied on pad surfaces.

Outer and inner surfaces of FGMs brake disk are considered ceramic-rich and metal-rich, respectively. The material of the pure pad is Aluminum, and the coefficient of friction *μ* between these materials is 1.4^31^.

### Finite element analysis by ANSYS software

#### Element type

**Figure 2:**
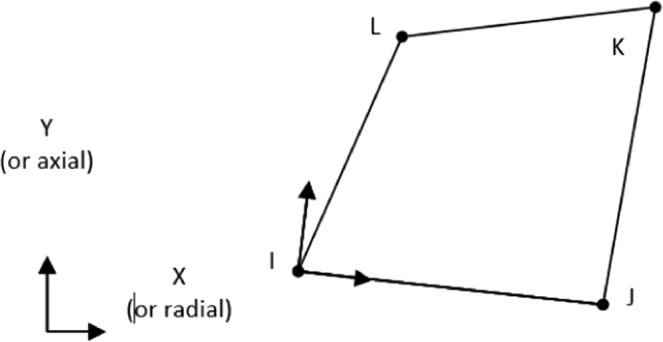
Geometry of PLANE13 Geometry of PLANE13.

PLANE13 with four nodes is employed like Shahzamanian *et al*.^31^. The shape function for axisymmetric and 2-D solid elements is quadrilateral solid without extra shape functions and mid-side node as described in Eq. (9) and shown in Fig. 2.

9 $$\begin{array}{c}u=\frac{1}{4}({u}_{I}\,(1-{s}_{1})(1-{s}_{2})+{u}_{J}\,(1+{s}_{1})(1-{s}_{2})+{u}_{K}\,(1+{s}_{1})(1+{s}_{2})+\\ \,{u}_{L}(1-{s}_{1})(1+{s}_{2})\,)\end{array}$$

where *u*, *u*_*I*_, *u*_*J*_, *u*_*K*_, and *u*_*L*_ represent motion and displacements of nodes I, J, K, and L, respectively and *s*_1_, and *s*_2_ are isoparametric variables.

#### Thermal loads

The thermal loads which are considered in FGMs rotating brake and pure pads disks are due to two sources. At first, it is considered that constant temperatures are applied at inner and outer surfaces of FGMs rotating brake disk. The second heat generation is between brake and pads.

It is assumed that the FGMs brake disk is rotated with constant angular velocity *ω*, and heat generation between the pad and brake disks is divided into two equal parts for pad and brake disk Shahzamanian *et al*.^31^.

### Thermo-mechanical relation

For thermo-mechanical relation, the cylindrical coordinate system (*r*, *θ*, *z*) is chosen. The geometry of thin FGMs brake disk, pads are considered as axisymmetric as well as loading parameters. The hollow FGMs brake disk with uniform thickness is considered, with ${r}_{i}$ and ${r}_{o}$ are inner and outer radii, respectively. The material of FGMs rotating brake disk changes from metal-rich to ceramic-rich consistently and smoothly from inner the surface to outer. The thermo-elastic relation by considering infinitesimal theory is discussed by Shahzamanian *et al*.^31^.

## Boundary Conditions

### Mechanical boundary condition for double side FGMs brake disk

Two mechanical parameters are more important for this investigation. First one is stress which occurs because of applying angular velocity to FGMs rotating brake disk, and it changes during the direction from maximum to zero at the outer surface. The second one is displacement that changes inversely of stress:

10 $${{\rm{u}}}_{{\rm{r}}}=0\,\mathrm{at}\,\,{\rm{r}}={{\rm{r}}}_{{\rm{i}}}$$

and

11 $${{\rm{\sigma }}}_{{\rm{r}}}=0\,{\rm{at}}\,{\rm{r}}={{\rm{r}}}_{{\rm{o}}}$$

### Thermal boundary condition for double side FGMs brake disk

The temperatures applied to the inner and outer surface of FGMs rotating brake disk and the heat generated due to friction are assumed for thermal boundary condition. Of course, the heat generated is considered to be divided between pad and brake disk equally. The contact areas are between ${r}_{i,pad}\le r\le {r}_{o}$ at *z* = 0 and *z* = *h* then for one side of brake disk and pad the heat, is calculated by using (12):

12 $${{\rm{Q}}}_{({\rm{Brake}}{\rm{and}}{\rm{Pad}})}=\frac{{{\rm{Q}}}_{{\rm{f}}}}{2}=\frac{{\rm{q}}\,\ast \,{\rm{\mu }}(r)\,\ast \,({\rm{r}}\,\ast \,{\rm{\omega }})}{2}$$

where *q* and ω are vertical pressure and angular velocity, respectively.

## Finite Element Approach for Contact Status

**Figure 3:**
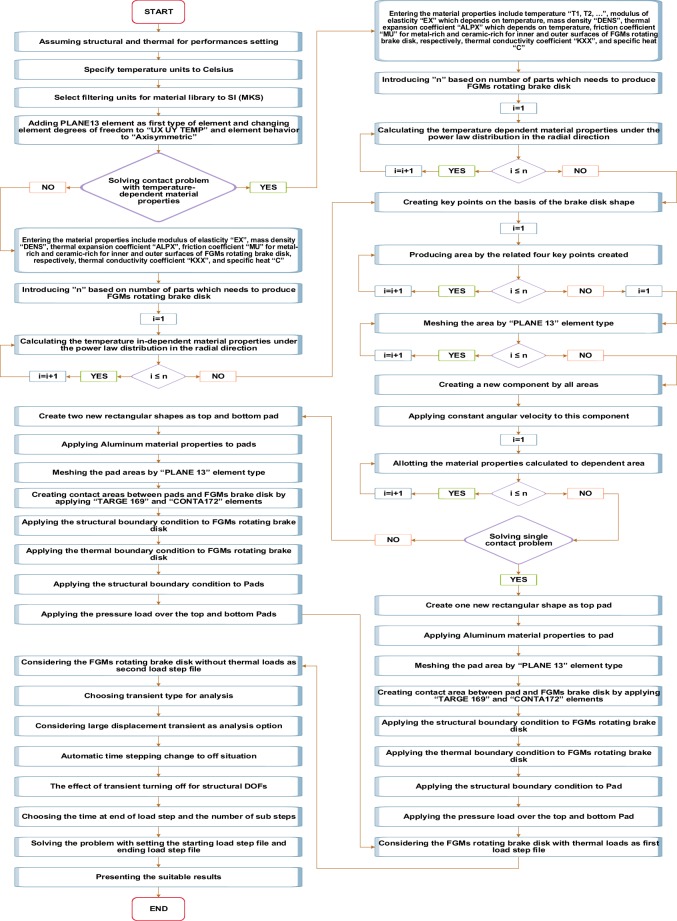
Flow chart of FEM scheme for temperature-dependent and temperature-independent FGMs brake disk.

The process of FEM analysis, which is done in the present study, has been presented in Fig. 3.

## Validation

For the verification of the presented results of this research, the numerical solution reported by Shahzamanian *et al*.^31^ for a FGMs brake disk with a constant value of contact line ratio (${R}_{cl}=\frac{{R}_{o,pad}-{R}_{{\rm{i}},pad}}{{R}_{o,disk}-{R}_{i,disk}}=0.25$), is employed. Shahzamanian *et al*.^31^ considered FGMs rotating brake disk with a single contact pad area with *Ro*, *pad* = *R*_*o*_, uniform vertical pressure *q* = 1000.0 *Kpa*, and constant angular velocity $\omega =1000\frac{rad}{s}$ while the ratio of pad thickness to FGMs brake disk thickness is assumed 0.66. Shahzamanian *et al*.^31^ demonstrated the vertical displacement of FGMs brake disk is between full-metal and full-ceramic brake disks.

**Figure 4:**
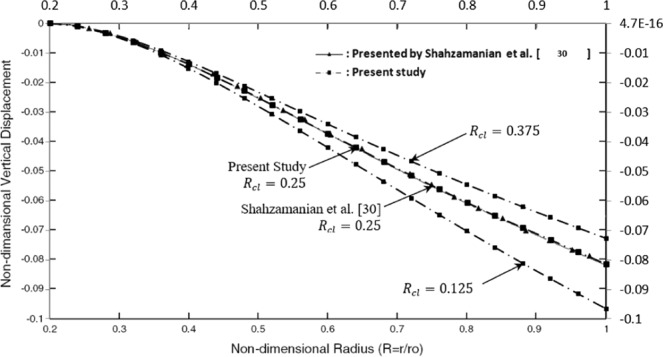
Non-dimensional vertical displacement against the non-dimensional radius.

The non-dimensional vertical displacement against radial direction for a constant value of grading index, *n* = 1.5 for different values of contact line ratio (*R*_*cl*_) is presented if Fig. 4. These results are obtained by employing FEM and compared with the special case with temperature-independent material properties and without any thermal load as considered by Shahzamanian *et al*.^31^.

As expected, the absolute vertical displacement increases from inner to the outer surface smoothly due to applying the same force to several contact line ratio. It can be noted that by increasing the value of ${R}_{cl}$, the absolute value of vertical displacement decreases. It can be seen that the results obtained in the non-dimensional form are in excellent agreement with the other results in the literature. Then the present approach is suitable for temperature-dependent material properties FGMs brake disk and double side contact problem.

## Numerical Results

For the numerical description of the thermo-elastic solution due to contact, thermal and bending loads, hollow fixed-free rotating FGMs brake disk with *h*_*Brake*_ = 10 *mm* and *r*_*o*_ = 5*r*_*i*_ is considered. Three diversity pad areas, as well as three cases namely one side contact area with temperature-independent and temperature-dependent material properties with mechanical and two sources of thermal loads and double side contact area with temperature-dependent material properties under thermo-mechanical load, are used. This study is conducted using Aluminum as inner-surface metal, Zirconia as outer-surface ceramic for FGMs rotating brake disk, and temperature-independent material properties are the same as presented by Shahzamanian *et al*.^31^ in Table 1.

**Table 1:** Temperature-independent Material Properties^31^.

Materials	*υ*	*ρ*(*kg*/*m*^3^)	*μ* (between pad and disk)	*k*(*W*/*m*°*C*)
Zirconia	0.3	5700	0.75	2.0
Aluminum	0.3	2700	1.40	209.0

The temperature-dependent material properties for Zirconia are from Shen^6^ and for Aluminum has been derived from McLellan and Ishikawa^38^ as listed in Table 2.

**Table 2:** Temperature-dependent Material Properties^6,38^.

Properties	Materials	*P*_0_	*P*_−1_	*P*_1_	*P*_2_	*P*_3_
E (Pa)	Zirconia	244.27e + 9	0	−1.371e-3	1.214e-6	−3.681e-10
	Aluminum	79.178e + 9	0	−3.882e-4	1.015e-7	−2.976e-10
*α*(K^−1^)	Zirconia	12.766e-6	0	−1.491e-3	1.006e-5	−6.778e-11
	Aluminum	1.238e-5	0	4.101e-3	−4.728e-6	2.456e-9

The FGMs rotating brake disk is considered to influence a body force due to constant angular velocity ($\omega =1000\,\frac{rad}{s}$), uniform vertical load (F = 282 MN), and thermal load (75 °C). The steady-state condition for the radial temperature at the inner surface of the brake disk made of Aluminum is 100 °C and the outer surface of the brake disk made of Zirconia at reference temperature 25 °C are considered. The coefficient of friction between FGMs rotating brake disk and pure pads is $\,0.75\le {\rm{\mu }}\le 1.4$.

Three different pad sizes with the dimensionless thickness ratio $\frac{\,{h}_{Pad}}{{h}_{brake}}=0.66$ are considered with contact line ratio is changed as:

13 $${R}_{cl}=1-\frac{{r}_{i,pad}}{{r}_{o,pad}}=1-0.1x\,x=7,\,\,8,\,\,9$$

where

14 $${r}_{o,pad}={r}_{o}$$

## Results and Discussions

The dimensionless results for displacement, strain, and stress are normalized and presented by $({\rho }_{Cr}{r}_{o}^{3}{\omega }^{2})/{E}_{cr}$, $\,{\rho }_{Cr}{r}_{o}^{2}{\omega }^{2}$, and $\,({\rho }_{Cr}{r}_{o}^{2}{\omega }^{2})/{E}_{cr}$factors, respectively.

### Single contact

#### Temperature-independent results

**Figure 5:**
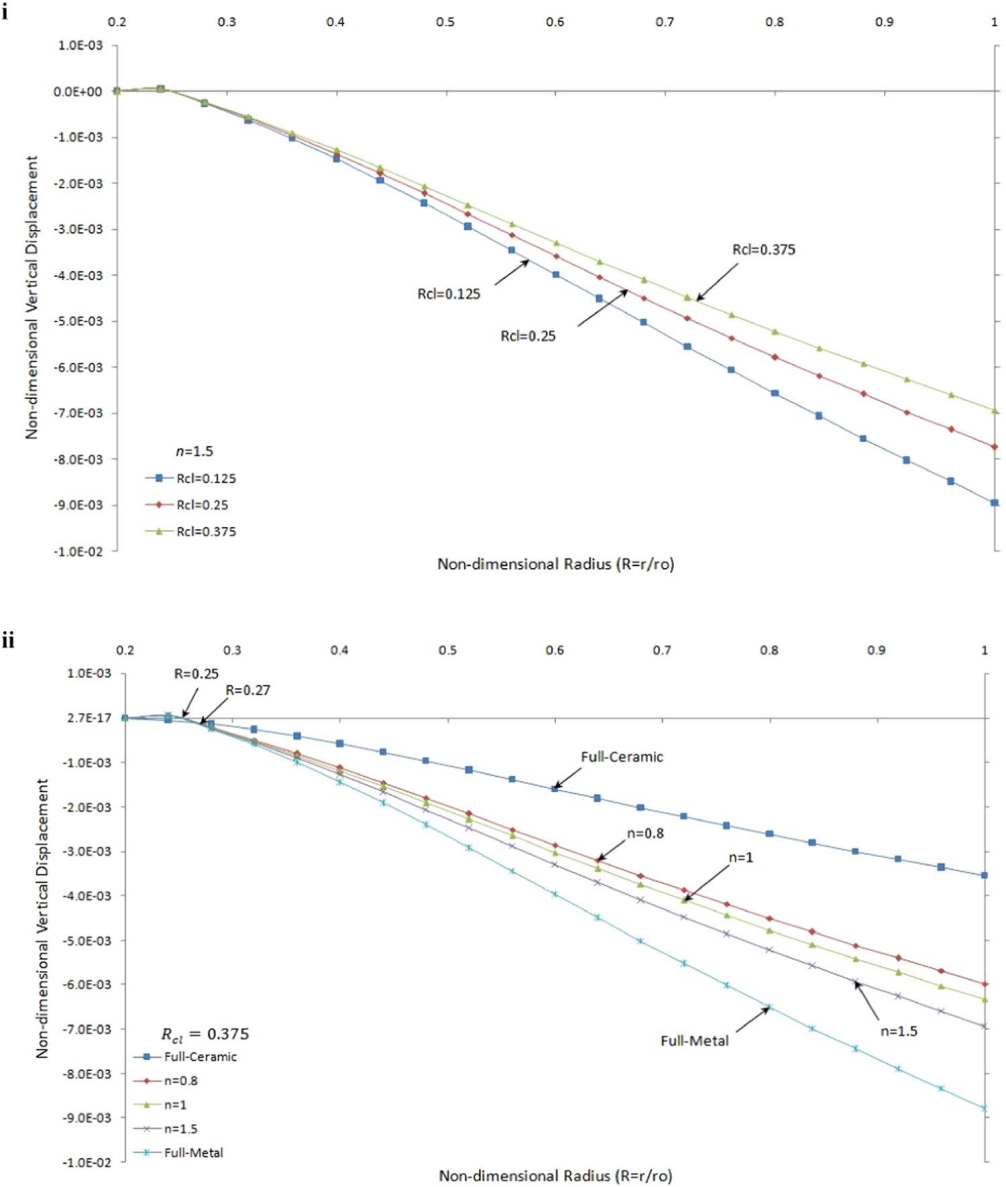
Non-dimensional vertical displacement versus the non-dimensional radius of one side contact with thermo-mechanical load: (i) in the FGMs rotating brake disk with different R_cl_ and n = 1.5 (ii) in the rotating brake disk with R_cl_ = 0.375 and different values of the grading index, n.

The non-dimensional vertical displacement versus the non-dimensional radius of one side contact with a thermo-mechanical load for different values of *R*_*cl*_ and the grading index, *n*, has been illustrated in Fig. 5.

Figure 5(i) shows the effect of the different value of contact line ratio (*R*_*cl*_) as given in Eq. (13) on the vertical displacement by fixing the value of *n* (*n* = 1.5). It is seen that the FGMs rotating brake disk with *R*_*cl*_ = 0.375 has smaller absolute vertical displacement compared to others. As expected greater value of *R*_*cl*_ makes a smaller value of displacement. In Fig. 5(ii), the effect of the grading index, *n*, on the vertical displacement with the constant value of *R*_*cl*_ is presented. As expected, the absolute values of non-dimensional displacement in the vertical direction for full-ceramic brake disk due to higher modulus of elasticity are less than those for the full-metal disk. It can be seen that the absolute non-dimensional vertical displacement decreases with the decrease of the grading index, *n*, value from $n\to \infty$ (homogeneous Aluminium brake disk) down to its minimum value to *n* = 0 (homogeneous Zirconia brake disk) where $\,0.27\le R\le 1$. The dimensionless vertical displacement of FGMs and full-metal brake disk go to the positive value close to the inner surface then change into negative values while it is thoroughly negative for full-ceramic brake disk. The slope of displacement increases with increasing the value of *n*.

**Figure 6:**
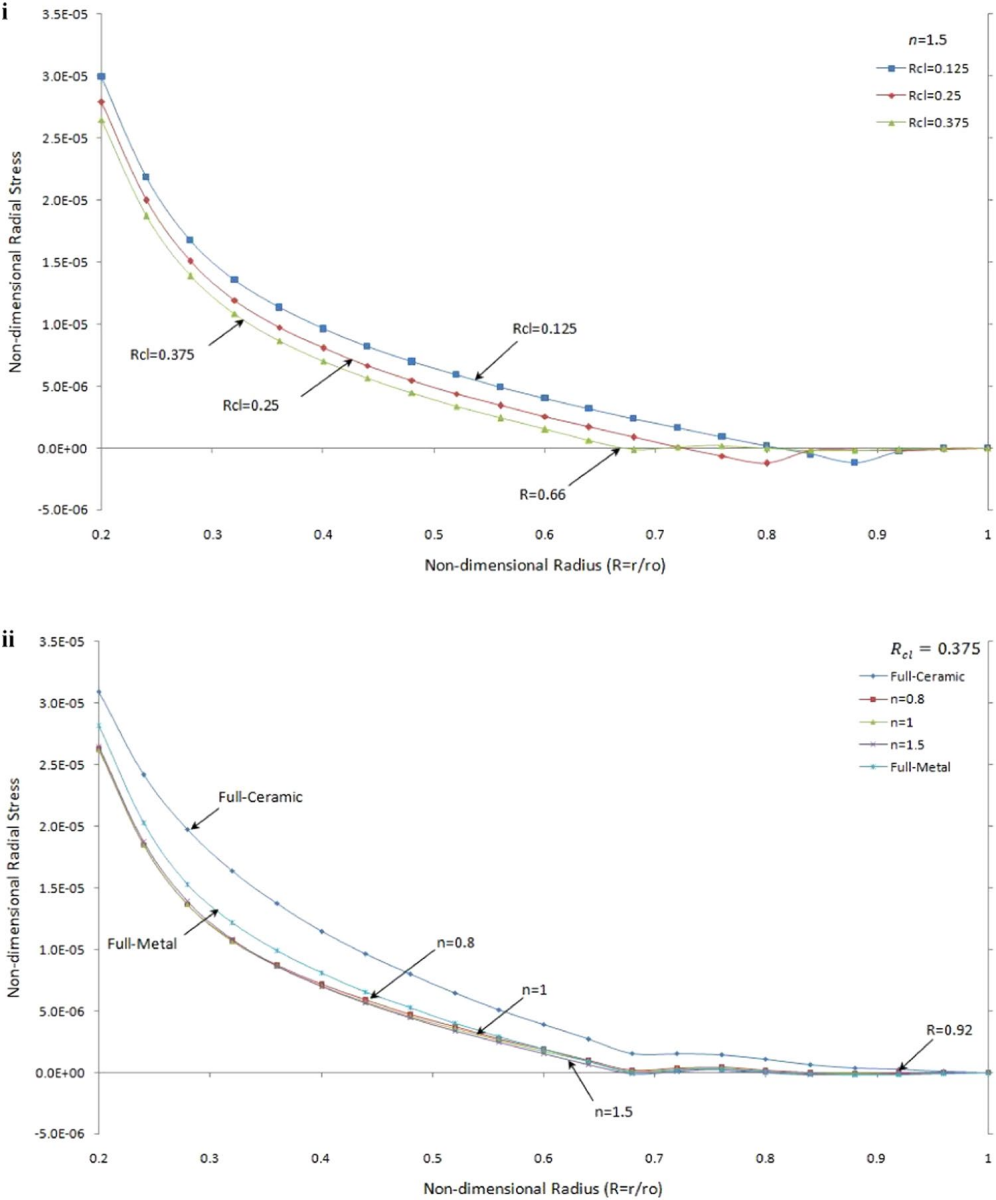
Non-dimensional radial stress versus the non-dimensional radius of one side contact with thermo-mechanical load (i) in the FGMs rotating brake disk with different R_cl_ and n = 1.5 (ii) in the rotating brake disk with R_cl_ = 0.375 and different values of the grading index n.

Figure 6 describes the non-dimensional radial stress against non-dimensional radius under thermo-mechanical load with different *R*_*cl*_ and grading index, *n*, under 75 °C thermal load respect to a reference temperature (25 °C) at the inner surface of rotating brake disk.

Figure 6(i) shows the effect of contact line ratio on the radial stress for the same value of the grading index when *n* = 1.5 and for different *R*_*cl*_. This graph recommends that the tensile radial stresses for *R*_*cl*_ = 0.375 is smaller than other *R*_*cl*_. It is observed that the radial stress at the inner surface of FGMs rotating brake disk with *R*_*cl*_ = 0.375 is the smallest, and also it takes zero value earlier than others. Moreover, it is seen that the variation of *R*_*cl*_ = 0.375 is narrower than others when $R\ge 0.66$. The influence of grading index, *n*, on the radial stress for rotating brake disk with the same value of *R*_*cl*_ is given in Fig. 6(ii). It is remarkable to mention that the radial stress for FGMs is smaller than full-metal and full-ceramic close to the inner surface. In contrast, the radial stress in the full-metal disk is smaller than that for FGMs brake disk where $R\ge 0.92$. As expected, the radial stress is zero at the outer surface.

**Figure 7:**
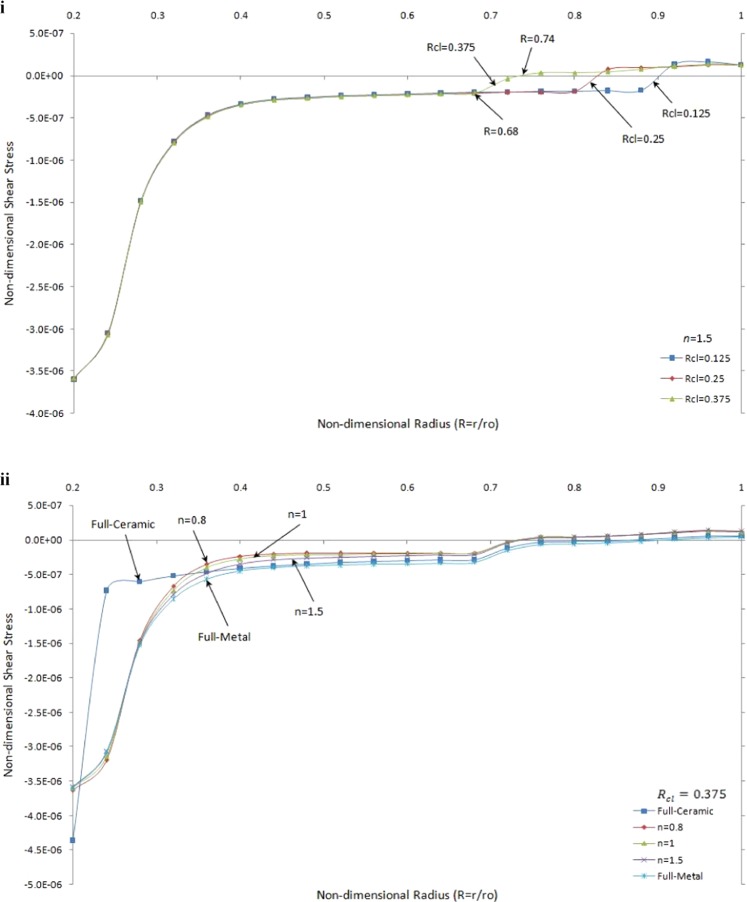
Non-dimensional shear stress versus the non-dimensional radius of one side contact with thermo-mechanical load (i) in the FGMs rotating brake disk with different R_cl_ and n = 1.5 (ii) in the rotating brake disk with R_cl_ = 0.375 and different values of the grading index n.

The variations of non-dimensional shear stress versus non-dimensional radius for a single contact situation and different value of *R*_*cl*_ and grading index *n* under thermo-mechanical load are shown in Fig. 7(i,ii).

Figure 7(i), presents the effect of *R*_*cl*_ on the shear stress for the same value of grading index *n = 1*.*5*. It is seen that the values of shear stresses are very close to each other where $R\le 0.68\,$for all value of *R*_*cl*_. It is noticed that for *R*_*cl*_ = 0.375 shear stress changes from the negative to the positive earlier than those for others where $(R\cong 0.74)$. It is seen that the maximum positive shear stress at smaller *R*_*cl*_ is greater than others as well as the slope of changing negative to positive shear stress. Figure 7(ii), demonstrates the effect of *n*, grading index, on the shear stress by considering the same geometry for pad disk. It is noticed that the absolute value of shear stress for full-ceramic is greater than that for full-metal and FGMs at the inner surface of brake disk while the absolute values of shear stress for FGMs brake disk are greater than pure material brake disks at outer surface. It is worth mentioning the positive shear stress always occurs after the first contact point $(R > 0.7)$.

**Figure 8:**
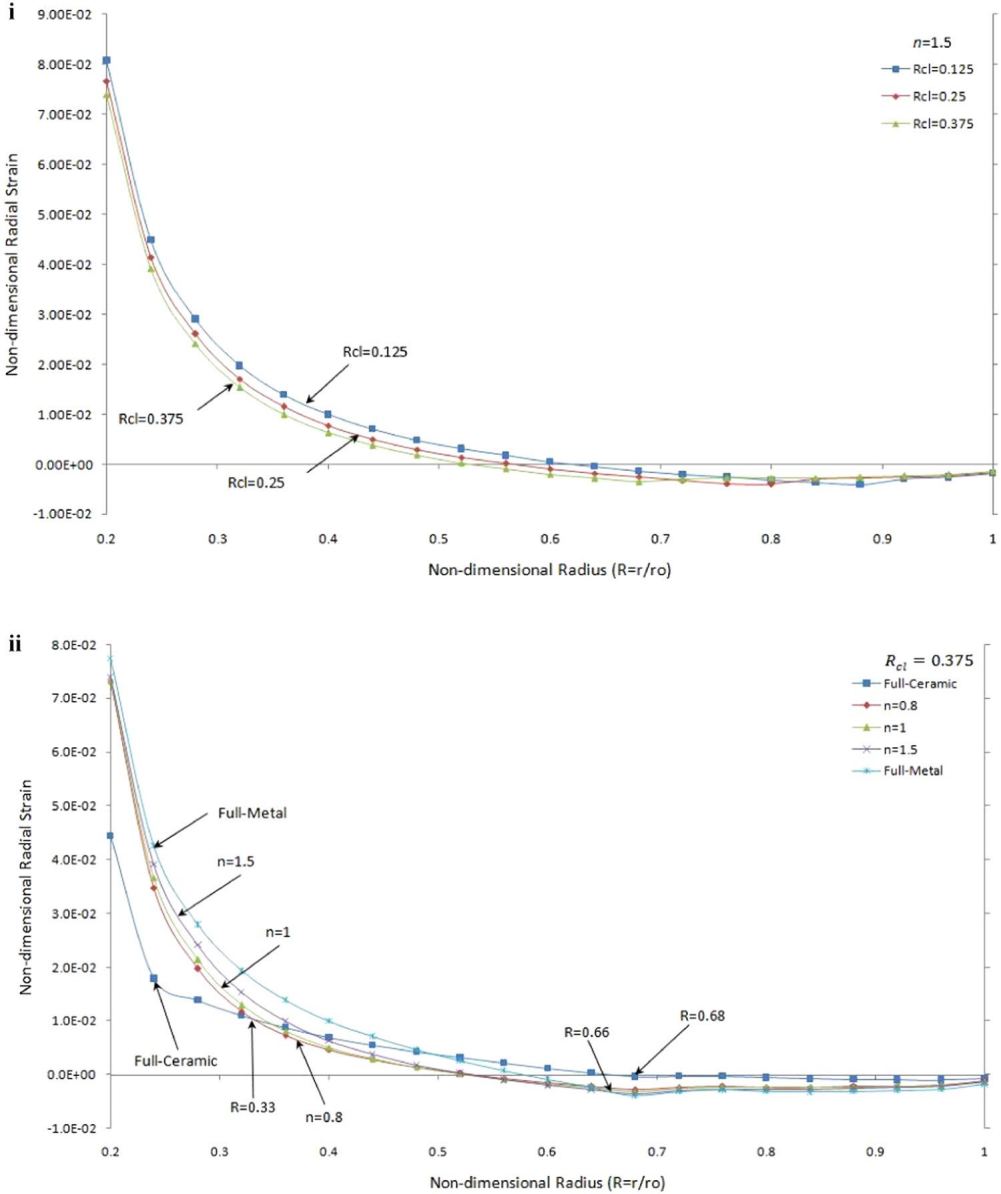
Non-dimensional radial strain versus the non-dimensional radius of one side contact with thermo-mechanical load (i) in the FGMs rotating brake disk with different R_cl_ and n = 1.5 (ii) in the rotating brake disk with R_cl_ = 0.375 and different values of the grading index n.

Figure 8(i,ii) present the non-dimensional radial strain versus non-dimensional displacement for single contact under thermo-mechanical load for different values of *n* and *R*_*cl*_.

It is seen in Fig. 8(i) that for the same value of *n* the maximum radial strain occurs at the inner surface of rotating brake disk and it changes smoothly to a negative value at the end of a brake disk. The radial strain before first contact point (*R* = 0.7) for *R*_*cl*_ = 0.375 is less than others; however, the radial strain converges to the approximately same value at the outer surface for all contact line ratios. For the same value of contact line ratio, Fig. 8(ii) shows that the full-metal has maximum absolute value and the full-ceramic has a minimum absolute value of radial strain while the FGMs radial strain occurs between them for different value of *n* for $R < 0.33\,$and $R > 0.66$. Also, the values of radial strains are negative for all brake disks close to the first contact point, for $R\ge 0.68$.

**Figure 9:**
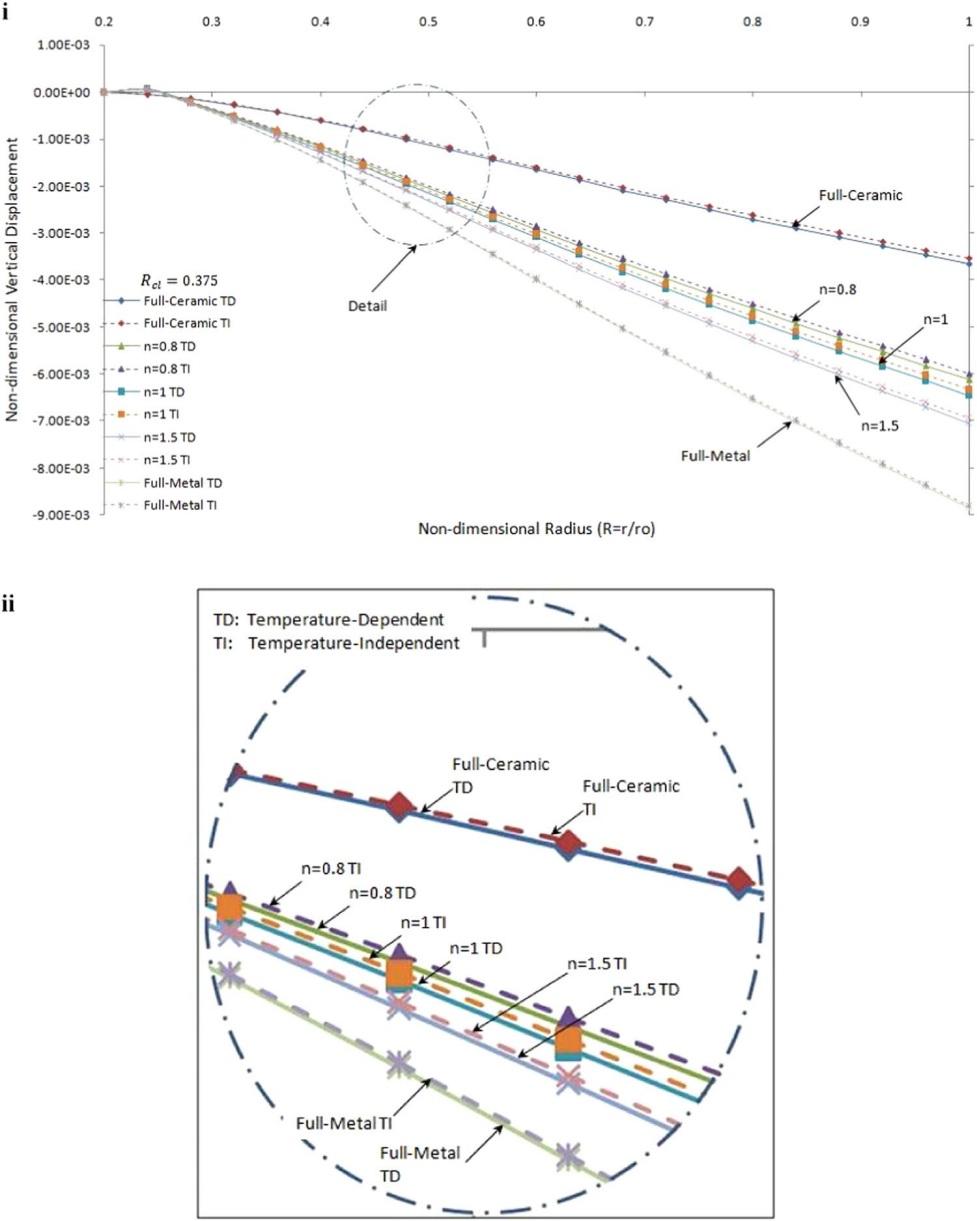
Non-dimensional vertical displacement versus the non-dimensional radius of one side contact with temperature-dependent and temperature-independent material properties under thermo-mechanical load: (i) in the FGMs rotating brake disk with R_cl_ = 0.375 and different values of the grading index n. (ii) detail of part (i).

Figure 9 compares the vertical displacement for temperature-dependent and temperature-independent material properties versus non-dimensional radius under thermo-mechanical loading for a specified value of R_cl_(R_cl_ = 0.375).

It is observed that the absolute values of vertical displacement in temperature-dependent material properties brake disks are greater than those for temperature-independent material properties brake disks. It is noticed that the difference between results for temperature-dependent with those for temperature-independent material properties decreases with the increases of grading index, *n*, in FGMs brake disk.

**Figure 10:**
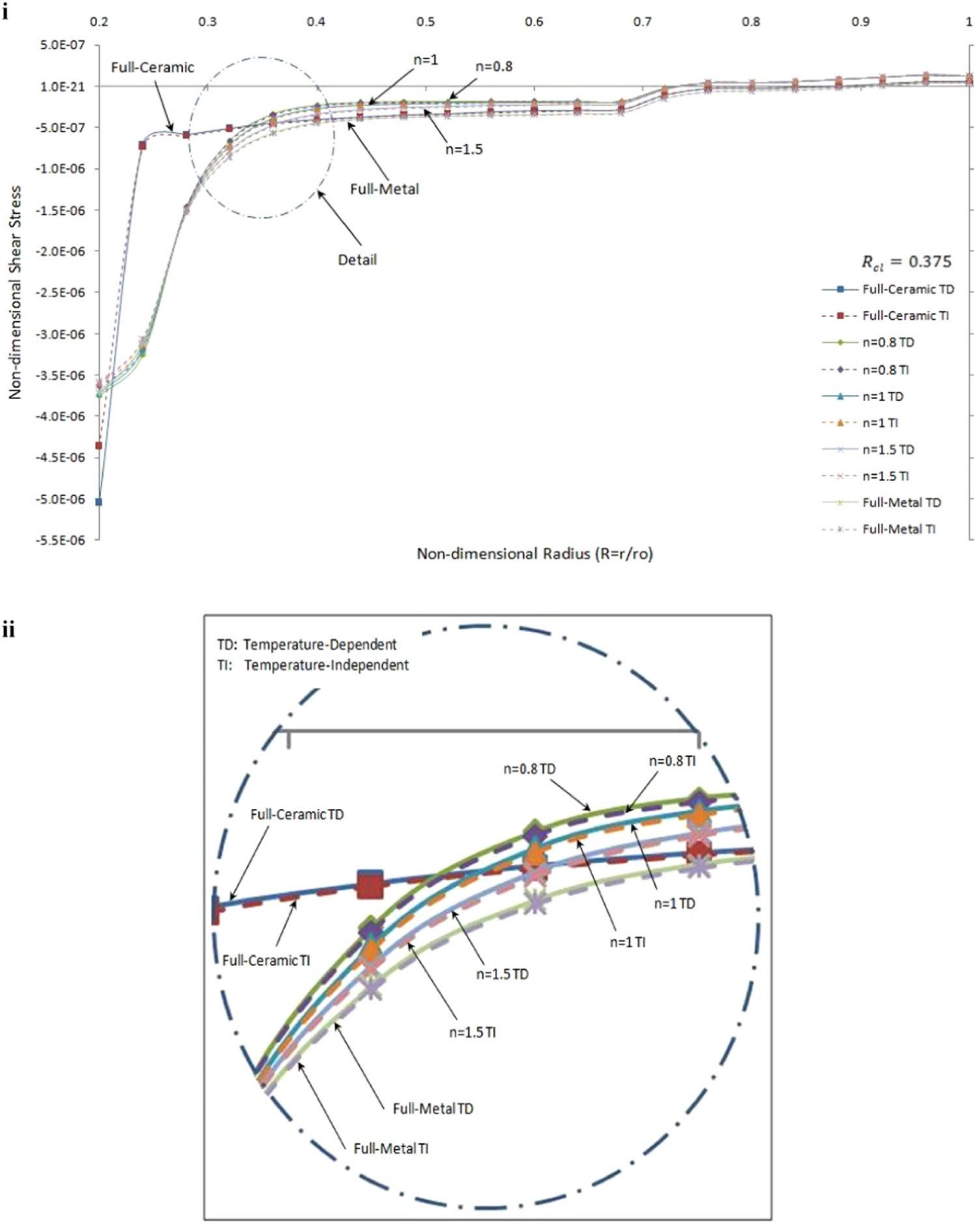
Non-dimensional shear stress versus the non-dimensional radius of one side contact with temperature-dependent and temperature-independent material properties under Rcl = 0.375 and different values of the grading index, n. (ii) detail of part (i).

Non-dimensional shear stress versus the non-dimensional radius of one side contact with temperature-dependent and temperature-independent material properties under thermo-mechanical load in the FGMs rotating brake disk for R_cl_ = 0.375 and different values of the grading index, *n*, are shown in Fig. 10.

It is seen that the absolute value of shear stress in temperature-dependent material properties brake disk are greater than those for temperature-independent material properties brake disk up to a specific radius. As expected, the trend is very similar to each other. Furthermore, the effect of temperature-dependent in full-ceramic brake disk is greater than those for the others at the inner surface. This phenomenon can be explained by the interaction between boundary condition and geometry.

Results from Figs 9 and 10 concluded that the thermo-mechanical response on a FGMs brake disk temperature-dependent material properties are more realistic. Therefore, for high-temperature thermal load, the temperature-dependent material properties should be considered to calculate the accurate thermo-mechanical response.

### Temperature-dependent double contact

**Figure 11:**
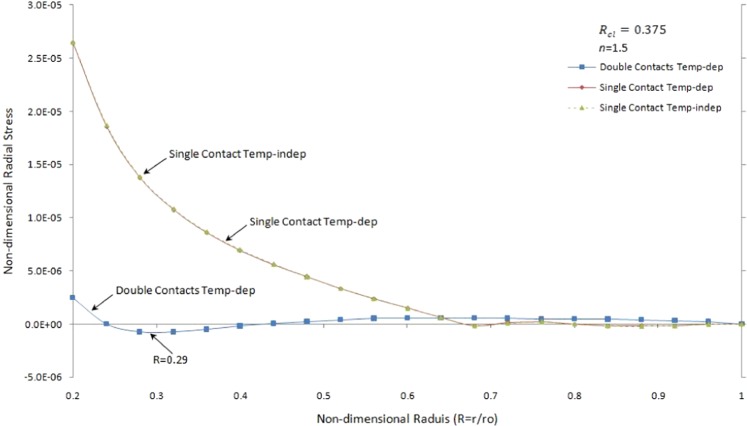
Non-dimensional radial stress versus the non-dimensional radius of one side contact with temperature-dependent and temperature-independent material properties and two side contacts under thermo-mechanical load in the FGMs rotating brake disk with R_cl_ = 0.375 and n = 1.5.

Figure 11 shows non-dimensional radial stress versus the non-dimensional radius of one side contact with temperature-dependent and temperature-independent material properties and two side contacts temperature-dependent material properties under thermo-mechanical load in the FGMs rotating brake disk with *R*_*cl*_ = 0.375 and *n* = 1.5.

**Figure 12:**
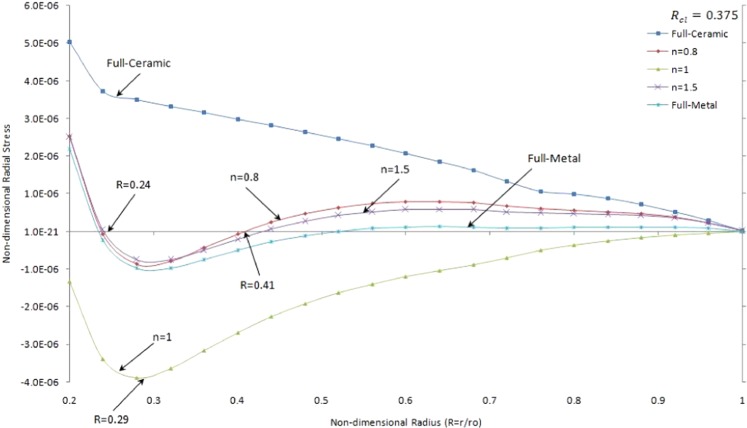
Non-dimensional radial stress versus the non-dimensional radius of two side contacts with temperature-dependent material properties under thermo-mechanical load in the FGMs rotating brake disk with R_cl_ = 0.375 and different values of the grading index, n.

The value of radial stress for temperature-independent and temperature-dependent material properties of single contact brake disk has the same behaviour while the behaviour of double contacts temperature-dependent material properties is completely different. Redial stress for pure brake disk and most of the FGMs brake disk start with tensile stress at the inner surface and tend to zero at the outer surface. It is worth to mention that the single contact brake disk with temperature-independent and temperature-dependent material properties have compressive and tensile values alternatively close to the outer surface. Moreover, the value of radial stress for double contacts brake disk can be converted from positive value to negative close to the inner surface. It is seen that the value of radial stress for double contacts brake disk with temperature-dependent material properties is tensile during contact line between the pad and brake disk (except for some specific value of grading index such as n = 1, see Fig. 12) unlike those for single contact brake disk. Also, the maximum value of compressive stress belongs to two sides contact brake disk with temperature-dependent material properties (*at R* = 0.29) while the maximum value of tensile stress is for one side contact with temperature-independent material properties (*at R* = 0.2).

Figure 12 presents non-dimensional radial stress versus non-dimensional radius of two side contacts with temperature-dependent material properties under thermo-mechanical load in the FGMs rotating brake disk with R_cl_ = 0.375 and different values of the grading index, *n*.

It is seen that the tensile stress and compressive stress through the disk belong to a full-ceramic brake disk and FGMs brake disk when n = 1, respectively. It is noticed that full-metal and FGMs brake disks with *n* = 0.8 *and* 1.5experience tensile stress close to inner and outer surfaces and compressive stress between $\,0.24 < R\le 0.41$. As expected, the values of radial stress tend to zero at the outer surface of the brake disk. By comparing the radial stress for one and double sides contact, it is seen that there is local minimum stress close to the inner surface for double contact while the variation of radial stress of single contact smoothly decreasing as shown in Fig. 6(ii). And also, by comparing the results of Figs 6 and 12, it is observed that there is tensile stress for single contact brake disk while there can be compressive stress for double sides contact brake disk where $\,0.2\le R\le 0.6$. Moreover, double contacts FGMs brake disk with *n* = 1 take compressive stress in Fig. 12, whereas it takes the tensile value as shown in Fig. 6(ii).

**Figure 13:**
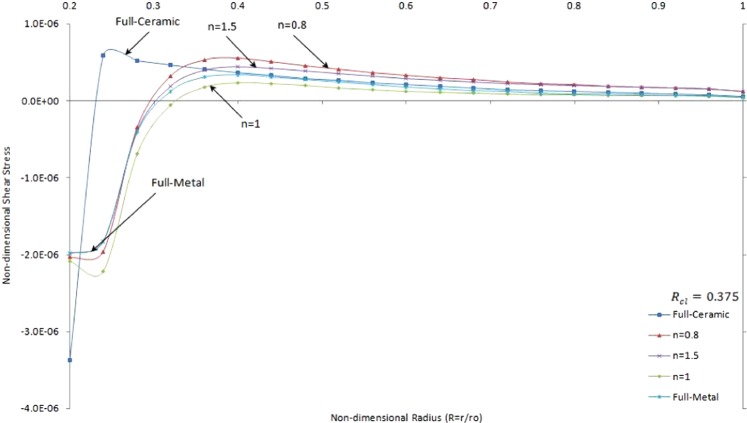
Non-dimensional shear stress versus the non-dimensional radius of two side contacts with temperature-dependent material properties under thermo-mechanical loads in the FGMs rotating brake disk with R_cl_ = 0.375 and different values of the grading index, n.

Figure 13 shows non-dimensional shear stress versus the non-dimensional radius of two side contacts with temperature-dependent material properties under thermo-mechanical loads in the FGMs rotating brake disk with R_cl_ = 0.375 and different values of the grading index, *n*.

It is seen that the shear stresses are negative close to the inner surface and then change to positive stress later on smoothly decrease to a very low positive value. It is noticed that the maximum and minimum shear stresses occur at full-ceramic brake disk. It is worth to mention that the positive shear stress is minimum for FGMs brake disk with n = 1. In addition, the slope of shear stress versus radius is positive for pure material brake disks while it is negative for specific FGMs brake disk (*n = 0*.*8* or *n = *1) close to the inner surface. It is seen that the positive shear stress for double side contact brake disk can occur close to inner surface oppose to results for single contact brake disk as shown Fig. 7(ii) and 13 while positive shear stress occurs only close to the outer surface due to the contact area.

**Figure 14:**
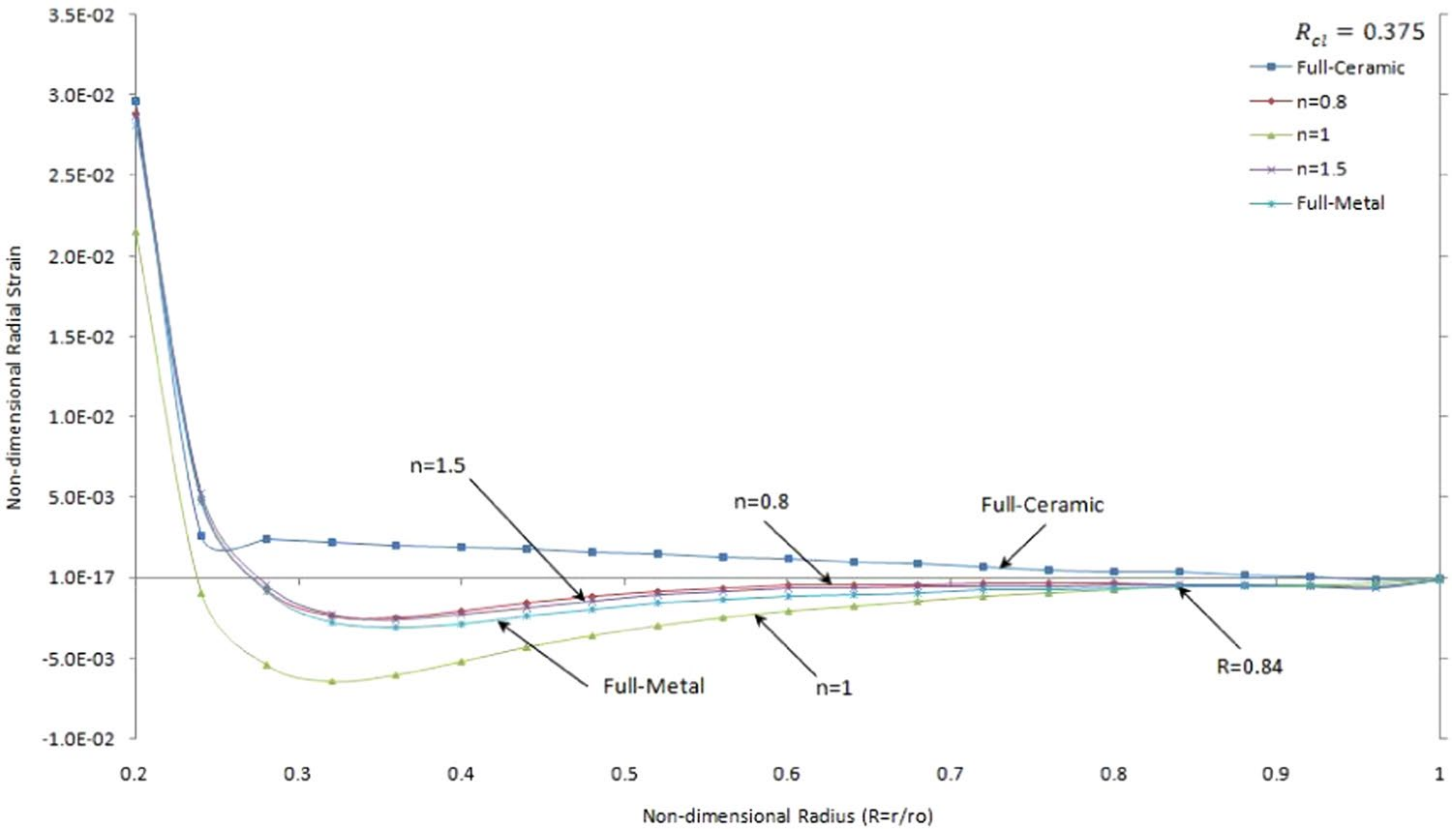
Non-dimensional redial strain versus the non-dimensional radius of two side contacts with temperature-dependent material properties under thermo-mechanical loads in the FGMs rotating brake disk with R_cl_ = 0.375 and different values of the grading index, n.

Figure 14 illustrates non-dimensional radial strain versus the non-dimensional radius of two side contacts with temperature-dependent material properties under thermo-mechanical loads in the FGMs rotating brake disk with R_cl_ = 0.375 and different values of the grading index, *n*.

It is seen that the slope of decreasing strain is sharp close to the inner surface and then smoothly decrease to zero value at the outer surface. There is a positive and negative radial strain for FGMs and full-metal brake disk whereas there is just positive radial strain for full-ceramic disk except close to the outer surface. As shown in Fig. 14 the results for FGMs brake disk for *n* = 1 is lower than other results where $R\le 0.84$. The interval of negative strain in double sides contacts brake disk is greater than those in one side contact brake disk as shown in Figs 8(ii) and 14, respectively. The absolute radial strains in single contact brake disks at the outer surface are greater than those for double contacts brake disks.

## Conclusions

The material properties of FGMs rotating brake disk change in radial direction under power-law distributions. The two thermal sources are dry contact friction and thermal load at inner and outer surfaces of FGMs brake disk. Aluminum as full-metal at inner surface and Zirconia as full-ceramic at the outer surface of rotating FGMs brake disk are considered. The constant vertical force is applied to three different pad areas. Thermo-elastic vertical displacement, radial stress, shear stress, and radial strain for single and double sides contact problem for FGMs rotating brake disk under specific boundary condition are obtained. The effects of single side and double sides contact related to different grading index, n, on the stress and strain fields are investigated, and results are compared.

The major conclusions of this investigation are:

1. The single contact FGMs brake disk under thermal-mechanical loads with R_cl_ = 0.375 has low tensile stress and absolute vertical displacement. The effect of contact line ratio is to shift the location and the value of zero stresses and vertical displacements.
2. The absolute values of vertical displacement in single contact FGMs brake disk due to thermo-mechanical loads remain between the maximum value for pure Aluminum brake disk and minimum value for pure Zirconia brake disk where $\,0.27\le R\le 1$. Contrary, the radial stresses for single contact FGMs brake disk are not between results for pure material disk along the radial direction.
3. Single contact FGMs brake disk with temperature-dependent material properties has larger absolute vertical displacement and smaller radial stress where $R < 0.6$, but with similar behavior due to thermo-mechanical load compared to those with temperature-independent material properties.
4. The radial stress for one side and two sides contacts with temperature-independent and temperature-dependent material properties start with tensile stress at inner surface except for some specific value of grading index (n = 1) for double contact, while, the behaviour of radial stress in single and double contact FGMs brake disk are totally different.
5. The value of radial stress for double contacts brake disk is tensile during contact line between pad and brake disk unlike those for single contact brake disk. Double contacts take the maximum value of compressive stress (*at R* = 0.29) while the maximum value of tensile stress is for one side contact with temperature-independent material properties (*at R* = 0.2).
6. The positive shear stress always occurs after the first contact point $(R > 0.7)$ for single contact while this phenomena in double contacts occurs close to the inner surface and then decrease to a positive value.
7. The maximum and minimum shear stresses for two sides contact occur at full-ceramic brake disk, unlike one side contact brake disk which occurs for FGMs brake disk.
8. The values of radial strain in a single contact are negative close to first contact point $R\ge 0.68\,$oppose to those for double contacts, which is negative close to the inner surface except for full-ceramic.
9. The absolute radial strains in double contact brake disks at the outer surface are less than those for single contact brake disks.

Results of this study suggest that the gradations of the constitutive components and temperature-dependent material properties, as well as contact line ratio, play an important role in the determination of thermo-mechanical responses in FGMs brake disks.
